# Design of a 2-Bit Neural Network Quantizer for Laplacian Source

**DOI:** 10.3390/e23080933

**Published:** 2021-07-22

**Authors:** Zoran Perić, Milan Savić, Nikola Simić, Bojan Denić, Vladimir Despotović

**Affiliations:** 1 Faculty of Electronic Engineering, University of Nis, Aleksandra Medvedeva 14, 18000 Nis, Serbia; zoran.peric@elfak.ni.ac.rs (Z.P.); bojan.denic@elfak.ni.ac.rs (B.D.); 2 Faculty of Sciences and Mathematics, University of Pristina in Kosovska Mitrovica, Ive Lole Ribara 29, 38220 Kosovska Mitrovica, Serbia; milan.savic1@pr.ac.rs; 3 Faculty of Technical Sciences, University of Novi Sad, Trg Dositeja Obradovica 6, 21000 Novi Sad, Serbia; 4 Department of Computer Science, Faculty of Science, Technology and Medicine, University of Luxembourg, Avenue de la Fonte 6, L-4364 Esch-sur-Alzette, Luxembourg; vladimir.despotovic@uni.lu

**Keywords:** image classification, Laplacian source, neural network, quantization

## Abstract

Achieving real-time inference is one of the major issues in contemporary neural network applications, as complex algorithms are frequently being deployed to mobile devices that have constrained storage and computing power. Moving from a full-precision neural network model to a lower representation by applying quantization techniques is a popular approach to facilitate this issue. Here, we analyze in detail and design a 2-bit uniform quantization model for Laplacian source due to its significance in terms of implementation simplicity, which further leads to a shorter processing time and faster inference. The results show that it is possible to achieve high classification accuracy (more than 96% in the case of MLP and more than 98% in the case of CNN) by implementing the proposed model, which is competitive to the performance of the other quantization solutions with almost optimal precision.

## 1. Introduction

Distributed neural networks, which process a lot of sensor data locally on edge devices instead of communicating with a cloud server, are becoming popular due to significantly reduced communication cost compared to a standard cloud offloading approach [1]. As utilization of server-grade graphics processing units (GPUs) in many embedded systems is impractical due to their enormous energy dissipation, there is a need to design resource-efficient systems for the deployment of various neural networks that are already trained [2]. The goal is to optimize design and resources in a such way that inference is only slightly degraded while there is significant energy saving. This goal can be commonly achieved by implementing various quantization techniques to simplify the numerical representation of weights, activations, and the intermediate results of convolution and fully connected layers, as well as to reduce their numerical ranges. We can highlight two approximation strategies that can be found in the literature: multilevel quantization and binarization. This way, the 32-bit floating-point representation of numerical values (i.e., full precision) can be reduced to lower representations.

Pioneering research focused on the effects of weight quantization in multilayer neural networks has been presented in [3,4,5]. Their main focus was to understand the performance degradation affected by weight quantization, including the convergence property of the learning algorithm [5]. However, quantization theory was significantly improved in later decades, and the contemporary understanding of an accurate quantizer design differs a lot. Although non-uniform quantization provides a better performance for a wide range of input signal variances [6,7] and advanced dual-mode asymptotic solutions are developed [8,9], simple uniform quantization [6,7,10,11,12] is the first choice when the simplicity of the system is one of the major goals. Thus, uniform quantization has been widely applied for quantizing parameters of neural networks (i.e., for neural network compression) [13,14,15,16,17,18], and different solutions have been considered, e.g., using 8-bits [13], 4-bits [14], or 2-bits [15,16,17,18]; further, non-uniform quantization has also been used [19,20,21]. It has been found that quantizing network parameters using 8-bits [13] or 16-bits [19] enable slightly lower performance when compared to the full precision case, mainly due to the ability of quantizers to achieve high quality reconstructed data. Further, in the case of applying quantizers with smaller resolution, e.g., with 4-bits [14] or 2-bits [15,16,17,18,20,21], performance degradation has been observed; however, the achieved results are still comparable, accompanied with a significantly high level of compression. Eventually, significant attention was paid to the development of binary quantizer models to compress neural networks [22,23,24,25,26], whose attractiveness lies in the amount of compression that can be achieved, with a goal to preserve competitive performance achievements.

In general, 2-bit quantization models require less energy compared to the models with a higher number of representative levels, which makes them appropriate for resource-constrained real-time systems. Consequently, we decided to focus on the designing of the simplest multilevel scalar quantizer model. The main contribution of this paper is the proposal of an accurate 2-bit optimal uniform quantizer design, achieved by optimizing step size or, equivalently, support region threshold (also known as the clipping factor). Optimization is carried out by considering the mean squared error (MSE) distortion, whereas the Laplacian source is assumed at the input. Specifically, the Laplacian source is widely used to model signals, such as speech [6,7,26,27] or images [6,7,26,28]; recent research conducted in [15,16,20,26,29] has shown its appropriateness in modeling the weights of neural networks. Note that the determination of the clipping factor for various quantizer solutions has been the subject of many research papers [14,15,16,26,29], implying the significance of this parameter. In addition to other research papers, e.g., [13,14,15,16,17,18,19,20,21,29], we perform several other analyses from the aspect of signal processing, including an analysis in the wide range of input signal variances and adaptation of the quantization model. 

We analyze the effectiveness of the proposed adaptive 2-bit quantizer in a real environment by implementing it in a neural network compression task, and the obtained performance is compared with the performance of the full-precision network, as well as with the performance of other contemporary 2-bit quantization models, either uniform [17,18] or non-uniform ones [20,21]. The first neural network model adopted in this paper is multi-layer perceptron (MLP) [30], which represents a kind of simple feedforward artificial neural network. Although it can be considered as a classical model and it is succeeded by the convolutional neural network (CNN) in advanced computer vision applications, its simplicity can be exploited in edge computing devices for real-time classification tasks [31,32,33,34]. We also employ a simple CNN network [30] for analysis, and both networks are used for image classification.

The rest of the paper is organized as follows: In Section 2, we describe in detail the proposed quantizer, including the design for the reference variance and analysis in a wide dynamic range. In Section 3, we provide the experimental results, obtained by implementing the considered quantizer in a neural network compression task. Finally, the advantages and disadvantages of the proposed model are summed up in the Conclusions Section.

## 2. A 2-Bit Uniform Scalar Quantizer of Laplacian Source

**Figure 1:**
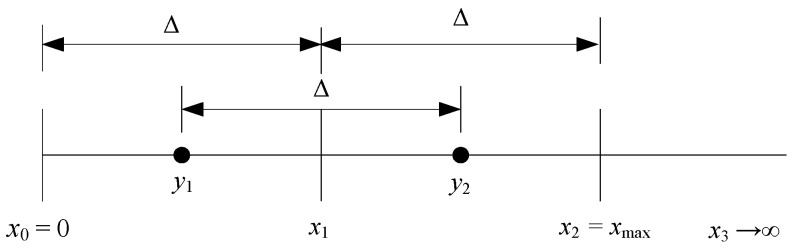
Illustration of the observed 2-bit uniform quantizer.

The 2-bit symmetrical uniform scalar quantizer we are interested in is illustrated in Figure 1. To uniquely define the quantizer, it is necessary to specify its parameters, namely the decision thresholds *x_i_* and the representative levels *y_i_* [6,7]. For such a uniform quantizer, it holds:

(1) $$x_{i}=i\Delta ,\;i=0,1,2$$

(2) $$y_{i}=\left(i-\frac{1}{2}\right)\Delta ,\;i=1,2$$

where Δ is the parameter known as the step size. In Figure 1, with $x_{\max}=x_{2}=2\Delta$ we denote the support region threshold of the quantizer (or equivalently the clipping factor). As the quantizer is symmetrical, parameters in the negative range are the inversions of the positive ones. Based on Equations (1) and (2), we can see that Δ (or $x_{\max}$) is a critical design parameter. The general manner to specify its value assumes the usage of some performance criterion, such as minimal MSE (mean squared error) distortion.

Let us define the designed-for and applied-to sources as the memoryless Laplacian with zero mean, described by probability density functions (PDFs) (3) and (4), respectively:

(3) $$q(x,\sigma_{q})=\frac{1}{\sqrt{2}\sigma_{q}}\exp\left(-\frac{\sqrt{2}\left|x\right|}{\sigma_{q}}\right)$$

(4) $$p(x,\sigma_{p})=\frac{1}{\sqrt{2}\sigma_{p}}\exp\left(-\frac{\sqrt{2}\left|x\right|}{\sigma_{p}}\right)$$

where $\sigma_{q}^{2}$ and $\sigma_{p}^{2}$ denote the variances.

In the following subsections, we consider the quantizer performance for two scenarios, namely the variance-matched ($\sigma_{q}^{2}=\sigma_{p}^{2}$) and variance-mismatched ($\sigma_{q}^{2}\neq \sigma_{p}^{2}$).

### 2.1. The Variance-Matched 2-Bit Uniform Quantizer

The variance-matched situation implies that the variance for which the quantizer is designed $\sigma_{q}^{2}$ and the variance of the input data to be quantized $\sigma_{p}^{2}$ are equal, and, accordingly, the equality of PDFs defined by (3) and (4) also holds. Therefore, we use $q(x,\sigma_{q})$ for the purpose of quantizer designing, and, further, we adopt $\sigma_{q}^{2}=1$, which is a commonly used approach in scalar quantization [6,7].

To measure the error produced within the data quantization process, MSE distortion is commonly used [7]. Considering Figure 1, we can see that the 2-bit uniform quantizer divides the range of the input data values into two regions, the inner defined in (−*x*_max_, *x*_max_) and the outer defined in (−∞,−*x*_max_) ∪ (*x*_max_, ∞). Therefore, the MSE distortion will be the sum of the distortions incurred in the inner (*D_in_*) and outer regions (*D_o_*), defined using the following lemmas:

**Lemma** **1.** *The inner distortion of a 2-bit uniform quantizer of Laplacian source depends on the quantization step ∆, and it is equal to*

(5) $$D_{in}=1-\frac{\Delta }{\sqrt{2}}+\frac{\Delta^{2}}{4}-\sqrt{2}\Delta \exp\{-\sqrt{2}\Delta \}-\left(1+\frac{\Delta }{\sqrt{2}}+\frac{\Delta^{2}}{4}\right)\exp\{-2\sqrt{2}\Delta \}\;$$

**Proof** **of** **Lemma** **1.** The inner distortion of an arbitrary quantizer *Q* with *N* representative levels for a processing signal described by an arbitrary source *p*(*x*) can be defined as [6,7]:

(6) $$D_{in}=\sum_{i=1}^{N}\int_{x_{i-1}}^{x_{i}}{\left(x-y_{i}\right)}^{2}p(x)dx$$

where ${\left\{x_{i}\right\}}_{i=0}^{N}$ are decision boundaries, whereas ${\left\{y_{i}\right\}}_{i=0}^{N}$ are representative levels. Let us consider that the source *p*(*x*) is the Laplacian of a unit variance and zero mean, i.e., let us *p*(*x*) = *q*(*x*, *σ_q_* = 1). For a 2-bit quantizer, we obtain:

(7) $$D_{in}=2\left(\int_{0}^{\Delta }{\left(x-y_{1}\right)}^{2}q(x,\sigma_{q}=1)dx+\int_{\Delta }^{2\Delta }{\left(x-y_{2}\right)}^{2}q(x,\sigma_{q}=1)dx\right)$$

Taking into account Equations (1)–(3), we obtain the following expression for the inner distortion:

(8) $$D_{in}=2\left(\int_{0}^{\Delta }{\left(x-\frac{\Delta }{2}\right)}^{2}\frac{1}{\sqrt{2}}\exp\{-\sqrt{2}x\}dx+\int_{\Delta }^{2\Delta }{\left(x-\frac{3\Delta }{2}\right)}^{2}\frac{1}{\sqrt{2}}\exp\{-\sqrt{2}x\}dx\right)$$

Finally, by solving integrals from the previous equation, we obtain the expression (5), which concludes the proof. □

**Lemma** **2.** *The overload distortion of a 2-bit uniform quantizer of Laplacian source depends on the quantization step ∆, and it is equal to*

(9) $$\;D_{o}=\left(1+\frac{\Delta }{\sqrt{2}}+\frac{\Delta^{2}}{4}\right)\exp\{-2\sqrt{2}\Delta \}\;$$

**Proof** **of** **Lemma** **2.** The overload distortion of an arbitrary quantizer *Q* for processing a signal described by an arbitrary source *p*(*x*) can be defined as [6,7]:

(10) $$D_{o}=2\int_{x_{\max}}^{+\infty }{\left(x-y_{\max}\right)}^{2}p(x)dx$$

where *x*_max_ is the support region threshold value, whereas *y*_max_ is the last representative level in the codebook. We observe the 2-bit uniform quantizer *x*_max_= 2∆, whereas *y*_max_ = 3∆/2. Thus, the overload distortion of the 2-bit uniform quantizer of Laplacian source is defined as:

(11) $$D_{o}=2\int_{2\Delta }^{+\infty }{\left(x-3\Delta /2\right)}^{2}q(x,\sigma_{q}=1)dx$$

By solving the previous integral, we obtain the expression for overload distortion defined with (9), concluding the proof. □

Based on Lemmas 1 and 2, the total distortion *D_t_* for the 2-bit uniform quantizer of Laplacian source is defined using the following expression:

(12) $$D_{t}=D_{in}+D_{o}=1-\frac{\Delta }{\sqrt{2}}+\frac{\Delta^{2}}{4}-\sqrt{2}\Delta \exp\{-\sqrt{2}\Delta \}$$

It can be noticed that distortion also depends on Δ, and its optimal value (denoted with Δ^opt^) is specified using the following lemma:

**Lemma** **3.** *The optimal value of Δ of a 2-bit uniform quantizer of Laplacian source can be determined using the following iterative rule:*

(13) $$\Delta^{\left(i+1\right)}=\sqrt{2}-\frac{\sqrt{2}}{2+\frac{1}{2}\exp\{\sqrt{2}\Delta^{\left(i\right)}\}},\;i=0,1,\ldots \;$$

**Proof** **of** **Lemma** **3.** Finding the first derivative of the total distortion (expression (12)) with respect to Δ and equaling it to zero, we obtain:

(14) $$\frac{\partial D_{t}}{\partial \Delta }=\frac{\Delta }{2}-\frac{1}{\sqrt{2}}+(2\Delta -\sqrt{2})\exp\{-\sqrt{2}\Delta \}=0$$

Based on the last equation, we can express Δ as:

(15) $$\Delta =\sqrt{2}-\frac{\sqrt{2}}{2+\frac{1}{2}\exp\{\sqrt{2}\Delta \}}$$

indicating that Δ can be determined iteratively, concluding the proof. □

As an appropriate initialization of the iterative process given with (13), one can use $\Delta^{\left(0\right)}=\frac{1}{\sqrt{2}}\ln4$ (motivated by the formula $x_{\max}=\sqrt{2}\ln N$ that was proposed in [35] as an approximate solution for *x*_max_ of *N*-levels uniform quantizer of Laplacian source). Moreover, by substituting this initial value into (13), one can obtain the asymptotic step size value:

(16) $$\Delta^{\left(1\right)}=\Delta^{a}=\frac{3}{4}\sqrt{2}$$

Such a determined asymptotic value can be useful if we want to quickly estimate the performance of the Laplacian 2-bit uniform quantizer (clearly, a more exact and accurate value for step size can be obtained using (13)). Let us define SQNR = 10∙log_10_(1/*D*), which is a standardly used objective performance measure of a quantization process [6,7]. Let SQNR(Δ*^a^* = 1.061) and SQNR(Δ^opt^ = 1.087) denote the SQNR obtained using the asymptotic and optimal step size value, respectively. It can be shown that these two SQNRs are very close, as the calculated relative error amounts to 0.08%, meaning that the proposed asymptotic step size is very accurate when compared to the optimal one. Nevertheless, the analysis conducted in this paper is focused only on the optimal 2-bit uniform quantizer of Laplacian source. Next, we will show that the minimum of the total distortion is achieved for Δ = Δ^opt^, as it is defined with the following lemma.

**Lemma** **4.** *Total distortion of a 2-bit uniform quantizer of Laplacian source is a convex function with a minimum at the point Δ = Δ^opt^.*

**Proof** **of** **Lemma** **4.** Second derivative of the total distortion is given by:

(17) $$\frac{\partial^{2}D_{t}}{\partial \Delta^{2}}=\frac{1}{2}+(4-2\sqrt{2}\Delta )\exp\{-\sqrt{2}\Delta \}$$

which also depends on Δ. On the other hand, the optimal value of Δ, i.e., Δ^opt^, is specified as (see Lemma 1):

(18) $$\Delta^{\mathrm{opt}}=\sqrt{2}-\frac{\sqrt{2}}{2+\frac{1}{2}\exp\{\sqrt{2}\Delta^{\mathrm{opt}}\}}$$

showing that the step size is upper bounded with $\sqrt{2}$, that is, 0 < Δ^opt^ < $\sqrt{2}$. Using this fact and applying it to (17), it holds that:

(19) $$\frac{\partial^{2}D_{t}}{\partial \Delta^{2}}{|}_{\Delta =\Delta^{\mathrm{opt}}}>0$$

which proves that distortion is a convex function, and the minimum is achieved at the point Δ = Δ^opt^. □

**Figure 2:**
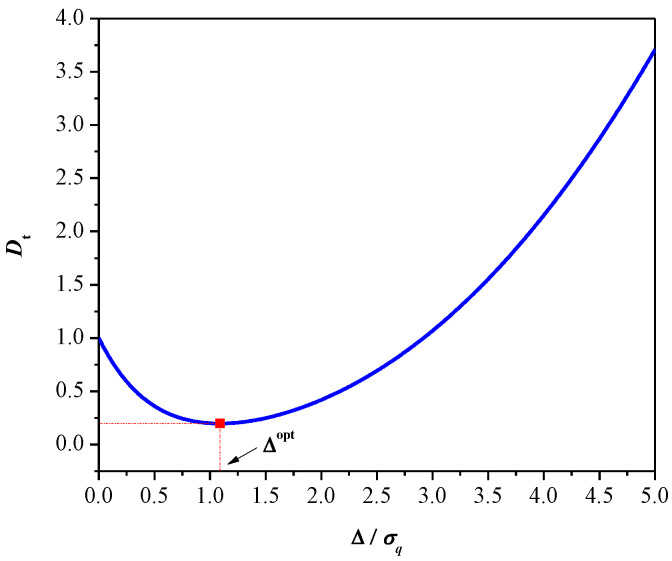
The total distortion depending on the parameter Δ for 2-bit uniform quantizer.

Figure 2 shows the total distortion with respect to Δ for the 2-bit uniform quantizer of Laplacian source obtained by numerical simulations, where perfect matching with the outcomes of Lemmas 3 and 4 is provided. 

### 2.2. The Variance-Mismatched 2-Bit Uniform Quantizer

The variance-mismatched scenario considered here implies the application of a 2-bit uniform quantizer, optimally designed in terms of MSE distortion for variance *σ_q_*^2^ = 1 (see Section 2.1), for processing the Laplacian data with variance *σ_p_*^2^, where it holds *σ_q_*^2^ ≠ *σ_p_*^2^. In particular, this scenario is worth investigating, as it is often encountered in practice and reveals the robustness level of the quantizer model, which is a very important property when dealing with non-stationary data [6,7]. On the other hand, it is known that the variance-mismatch effect may cause serious degradation in quantizer performance [6,7,36,37]. In this subsection, we derive the closed-form expressions for the performance evaluation of the discussed quantizer. 

As in the previous subsection, performance of the variance-mismatched 2-bit uniform quantizer is investigated using MSE distortion or, equivalently, using SQNR. Total distortion can be assessed as follows:

(20) $$\begin{array}{ll} D_{t} & =2\left(\int_{0}^{\Delta (\sigma_{q})}{\left(x-\frac{\Delta (\sigma_{q})}{2}\right)}^{2}p(x,\sigma_{p})dx+\int_{\Delta (\sigma_{q})}^{+\infty }{\left(x-\frac{3\Delta (\sigma_{q})}{2}\right)}^{2}p(x,\sigma_{p})dx\right)\\ & =\sigma_{p}^{2}+\frac{\Delta^{2}(\sigma_{q})}{4}-\sigma_{p}\frac{\Delta (\sigma_{q})}{\sqrt{2}}\left(1+2\exp\left\{-\frac{\sqrt{2}\Delta (\sigma_{q})}{\sigma_{p}}\right\}\right) \end{array}$$

where Δ(*σ_q_*) = *σ_q_* Δ denotes the optimal step size value determined for variance *σ_q_*^2^ = 1 (see Section 2.1). 

Let us define the degree of mismatch *ρ* = *σ_p_*/*σ_q_* [36]. Then, total distortion becomes:

(21) $$D_{t}=\sigma_{p}^{2}\left(1+\frac{\Delta^{2}}{4\rho^{2}}-\frac{\Delta }{\sqrt{2}\rho }\left(1+2\exp\left\{-\frac{\sqrt{2}\Delta }{\rho }\right\}\right)\right)$$

SQNR can be calculated according to:

(22) $$SQNR(\rho )=10\log_{10}\left(\frac{\sigma_{p}^{2}}{D_{t}(\rho )}\right)=10\log_{10}\left(\frac{1}{1+\frac{\Delta^{2}}{4\rho^{2}}-\frac{\Delta }{\sqrt{2}\rho }\left(1+2\exp\left\{-\frac{\sqrt{2}\Delta }{\rho }\right\}\right)}\right)$$

**Figure 3:**
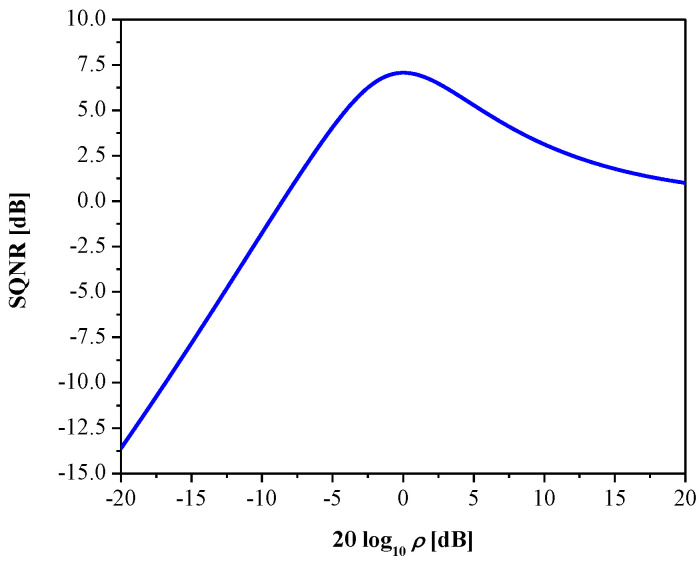
SQNR of 2-bit uniform quantizer (designed optimally with respect to MSE distortion) in a wide dynamic range of input data variances.

In Figure 3, we show SQNR as the function of *ρ* for the proposed quantizer. Observe that the SQNR curve attains its maximal value of 7.07 dB for the variance-mismatch case (*σ_p_* = *σ_q_* = 1, that is, *ρ* = 1), but it does not retain that value over the entire range and significantly decreases. Accordingly, the robustness of the quantizer is not at the satisfactory level, as the variance-mismatch effect has a strong influence on its performance; this, in turn, is reflected in limited efficiency of processing various Laplacian data. 

In a real situation, such as the quantization of neural network parameters, the convergence of the model depends on several aspects, including the dataset size, network architecture, number of epochs etc.; therefore, differences between designed-for and applied-to sources may exist. In particular, the decreasing of SQNR (note that we deal with low-resolution quantization where SQNR values are rather small) can be a serious issue, as it may have negative effects on classification accuracy, which is undesirable. Furthermore, the mentioned effect is also present even in the case of high-resolution (*N* is high) quantization, as pointed out in [38], where the post-quantization of neural network weights is performed. Hence, it is of particular interest to avoid variance-mismatch and enhance performance of the quantizer by achieving constant SQNR across a wide variance range of input data. To this end, we describe an efficient method that is based on adaptive quantization, which can also be important for the final deployment.

### 2.3. Adaptation of the 2-Bit Uniform Quantizer

**Figure 4:**
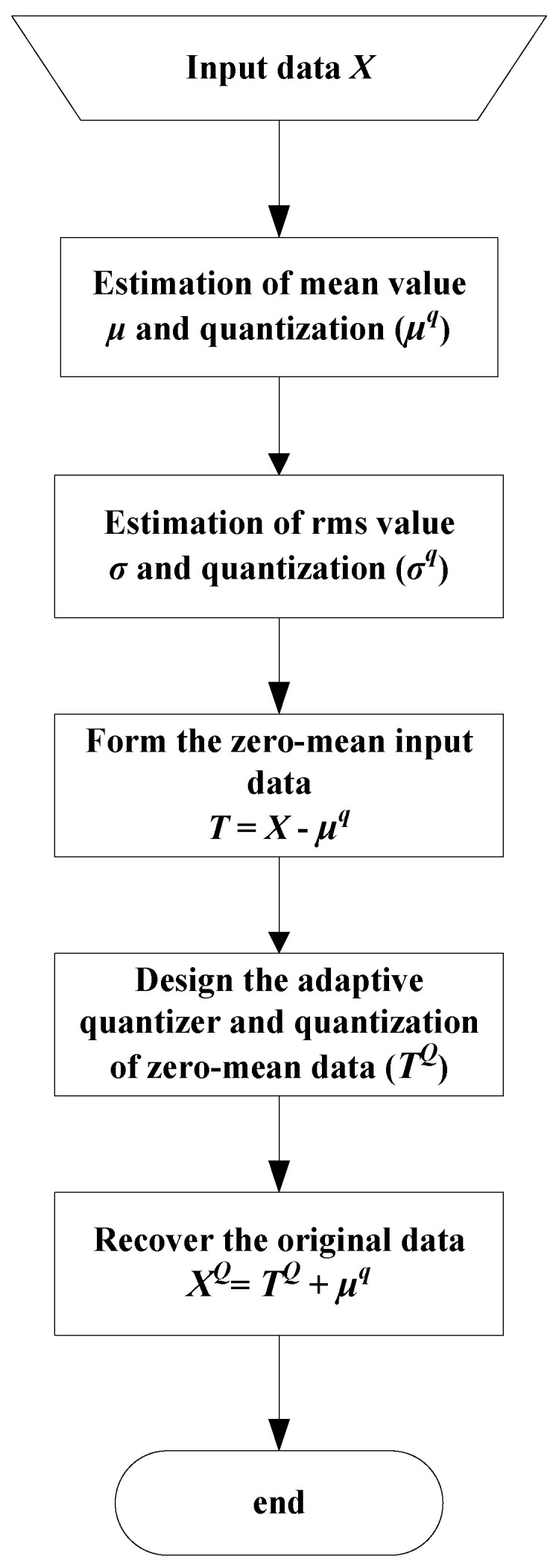
Adaptation process of 2-bit uniform scalar quantizer.

The goal of this subsection is to make the proposed quantizer able to provide improved performance expressed by a constant SQNR over the variance range of interest. This can be achieved using an adaptation technique [6,7], where some statistical parameters, e.g., variance and mean, are estimated from the input data and further used for adaptation purposes. Let us denote with *x_i_* the data of the input source *X*, where *i* = 1, …, *M*, and *M* is the total number of data samples. A flowchart is depicted in Figure 4 and can be described with the following steps:

**Step 1. Estimation of the mean value and quantization.** The mean value of the input data can be estimated as [6,7]:

(23) $$\mu =\frac{1}{M}\sum_{i=1}^{M}x_{i}$$

This parameter is quantized using a floating-point quantizer [39] and stored using 32 bits (32-bits floating point format is typically used in neural network applications [13,14,15,16,17,18,19,20,21,22,23,24,25,26,29,30]). 

**Step 2. Estimation of the standard deviation (rms value) and quantization.** The rms of the input data can be evaluated according to [6,7]:

(24) $$\sigma =\sigma_{p}=\sqrt{\frac{1}{M}\sum_{i=1}^{M}{\left(x_{i}-\mu \right)}^{2}}$$

This parameter is also quantized using a 32-bits floating-point quantizer [39]. 

**Step 3. Form the zero mean input data.** Each element of the input source *X* is reduced by the quantized mean, and zero mean data denoted with *T* are obtained:

(25) $$T=X-\mu^{q}$$

where *μ^q^* is the quantized version of *μ*. Note that this is carried out in order to properly use the quantizer (as it is designed for a zero mean Laplacian source). 

**Step 4. Design of adaptive quantizer and quantization of zero mean data.** The quantized variance, *σ^q^*, is used to scale the crucial design parameter Δ as follows:

(26) $$\Delta \left(\sigma_{p}\right)=\left(1+\varepsilon \right)\sigma^{q}\Delta \left(\sigma_{q}\right)$$

and the adaptive quantizer is obtained, where *ε* is a constant used to compensate the imperfections between the theoretical model and the distribution of the experimental data. Input data *t_i_* of the source *T* are passed through the adaptive quantizer, and the quantized data *t_i_^q^* are obtained. 

**Step 5. Recover the original data.** Since the mean value is subtracted from the original data and further quantized (using 32 bits), an inverse process has to be performed to recover the original data:

(27) $$x_{i}^{Q}=t_{i}^{q}+\mu^{q},\;i=1,\ldots ,M$$

where *x_i_^Q^* denotes the data recovered after quantization. It should be emphasized that the described process is equivalent to the normalization process widely used in neural network applications [15,18,22], as the same performance in terms of SQNR can be achieved [40]. Particularly, the normalization process assumes the following steps:

**Step** **1.** **Estimation of the mean value and quantization.**
**Step** **2.** **Estimation of the standard deviation (rms value) and quantization.**
**Step** **3.** **Normalization of the input data.** Each element of the input source *X* is normalized according to:
  (28) $$T=\frac{X-\mu^{q}}{\sigma^{q}\left(1+\varepsilon \right)}$$
  and the source *T* with transformed (normalized) coefficients is formed.
**Step** **4.** **Quantization of the normalized data.** To quantize normalized data (modeled as the PDF with zero mean and unit variance), the quantizer designed in Section 2.1 can be used, and quantized data *t_i_^q^* are obtained.
**Step** **5.** **Denormalization of the data.** Since the input data are appropriately transformed for the purpose of efficient quantization, an inverse process referred to as denormalization has to be performed to recover the original data:
  (29) $$x_{i}^{Q}=t_{i}^{q}\sigma^{q}+\mu^{q},\;i=1,\ldots ,M$$

To measure the theoretical performance of the adaptive 2-bit uniform scalar quantizer, we can also use Equation (22) under the constraint that Δ is replaced with Δ(*σ_p_*) defined with (26), which gives:

(30) $$\begin{array}{ll} \mathrm{SQNR} & =10\log_{10}\left(\frac{1}{1+\frac{\Delta^{2}\left(\sigma_{p}\right)}{4\rho^{2}}-\frac{\Delta \left(\sigma_{p}\right)}{\sqrt{2}\rho }\left(1+2\exp\left(-\frac{\sqrt{2}\Delta \left(\sigma_{p}\right)}{\rho }\right)\right)}\right)\\ & \approx 10\log_{10}\left(\frac{1}{1+\frac{{\left(1+\varepsilon \right)}^{2}\Delta^{2}}{4}-\frac{\left(1+\varepsilon \right)\Delta }{\sqrt{2}}\left(1+2\exp\left(-\sqrt{2}\left(1+\varepsilon \right)\Delta \right)\right)}\right) \end{array}$$

since $\sigma^{q}=\sigma_{p}^{q}\approx \sigma_{p}$, as we use a high number of bits for its quantization.

**Figure 5:**
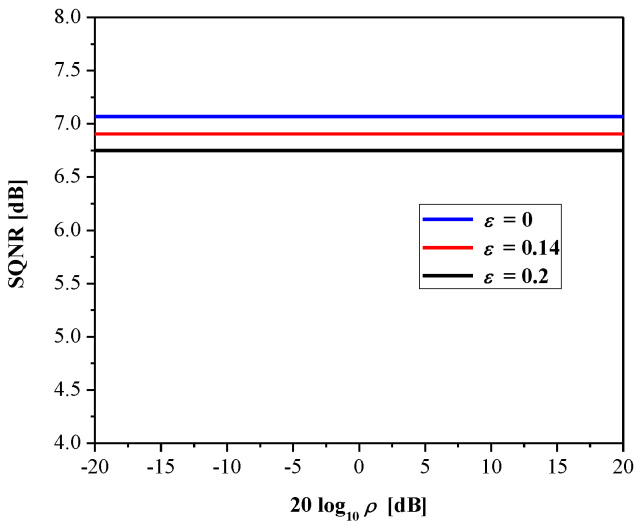
SQNR of the adaptive 2-bit uniform quantizer in a wide dynamic range of input data variances.

Figure 5 plots the SQNR of the adaptive 2-bit uniform quantizer, where it is obvious that adaptation successfully improves performance when compared to the case observed in Section 2.2 (see Figure 3), since a constant SQNR value is achieved in the considered range (that is, SQNR is independent of the input data variance). Note also the influence of parameter *ε* on the performance, where the case *ε* = 0 implies perfect adaptation of the quantizer to the data variance and the achieved SQNR is equal to 7.07 dB (this value corresponds to the optimal 2-bit uniform quantizer). With the increasing of *ε*, performance becomes slightly lower, as adaptation is not perfect. 

## 3. Experimental Results and Discussion

This section investigates the suitability of 2-bit uniform quantization in the compression of neural networks. Firstly, we consider the MLP network architecture [30] applied to an image classification task and investigate how the quantization of weights affects performance of the network measured by classification accuracy. Specifically, MLP is still attractive and is applied in solving different challenges occurring in different research areas, e.g., [30,31,32,33,34], and, hence, it is worth investigating. Further, the results from the aspect of SQNR will also be analyzed by checking the agreement between the theoretically and experimentally obtained values.

The MLP network used in the experiment is constituted by the input, hidden, and output layer. Training, validation, and test data are taken from the MNIST database [41], which contains 70,000 grayscale images of handwritten single digits with a resolution of 28 × 28 pixels, where 60,000 and 10,000 images are intended for training and testing purposes, respectively. We apply the rectified linear unit (ReLU) activation function in the hidden layer and softmax activation function in the output layer. We also perform the following setup: regularization rate = 0.01, learning rate = 0.0005, and batch size = 128. 

**Figure 6:**
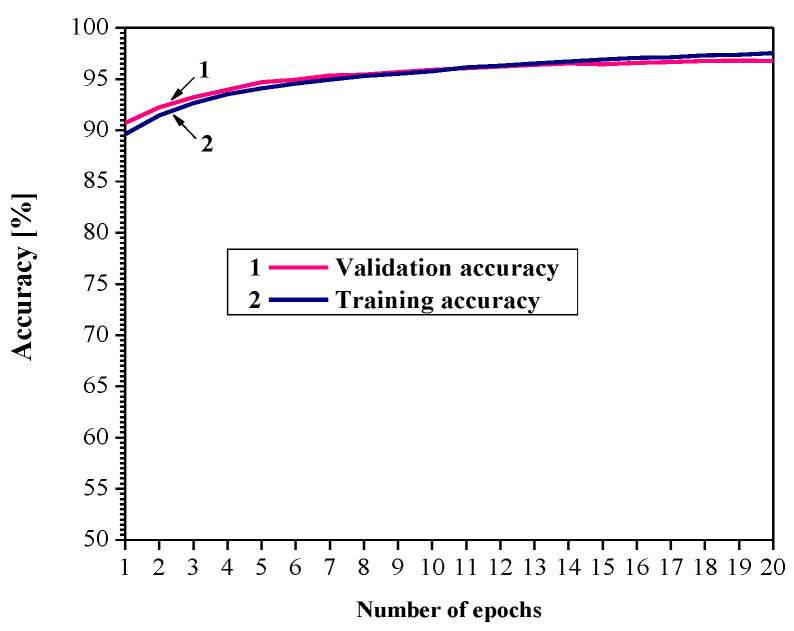
Learning curves for MLP neural network.

In our consideration, the goal is to apply an adaptive 2-bit uniform quantizer to quantize the weights of a trained MLP network, that is, to perform post-training quantization. Thus, Figure 6 shows the learning curves for the employed network, where after 20 epochs we obtain a training accuracy of 97.37%. As our model is evaluated on the training dataset and on a hold-out validation dataset after each update during the training, we show the measured performance by drawing two learning curves (training and validation learning curves). In this case, training and validation accuracy increase to a point of stability and have a minimal gap between their values, so that overfitting and underfitting do not exist.

**Figure 7:**
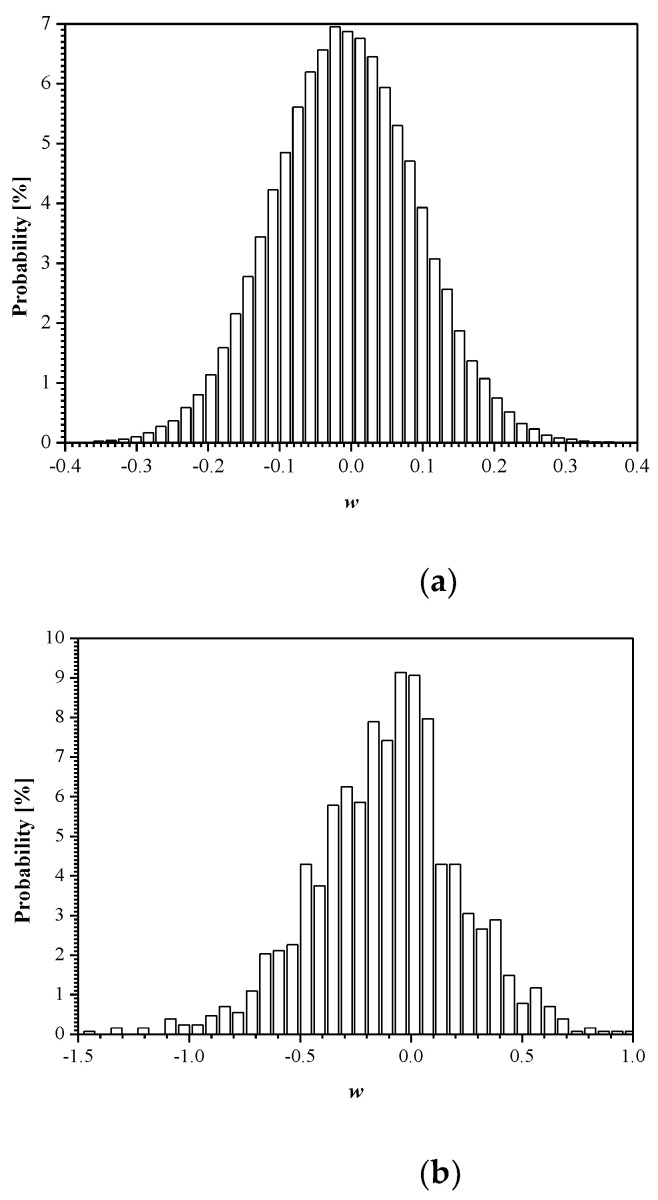
Distribution of weights of trained MLP network: (**a**) between input and hidden layer and (**b**) between hidden and output layer.

In Figure 7, we present the histograms for the weights both between the input and hidden layer (784 × 128 = 100,352 in total) and between the hidden and output layer (128 × 10 = 1280 in total) of the trained MLP network (training is completed at the 20th epoch). Note also that there is a significantly lower number of weights between the hidden and output layer, and, hence, there is little benefit to compress them. It should be noted that the good approximation of the distribution given in Figure 7a is the Laplacian distribution with some specific value *σ_w_*^2^ and mean value *μ_w_* that is very close to zero. This, in turn, enables proper implementation of the developed adaptive quantizer model (Section 2.3).

Let us further define SQNR*^ex^*, by which the experimental value of SQNR can be measured:

(31) $${\mathrm{SQNR}}^{ex}=10\log_{10}\left(\frac{\sigma_{w}^{2}}{D_{{}^{w}}}\right)=10\log_{10}\left(\frac{\frac{1}{W}\sum_{i=1}^{W}w_{i}^{2}}{\frac{1}{W}\sum_{i=1}^{W}{\left(w_{i}-w_{i}^{q}\right)}^{2}}\right)$$

where *D_w_* is the distortion inserted by the adaptive uniform quantization (using 2-bits) of weights, *W* is the total number of weights, and *w_i_* are original while *w_i_^q^* are quantized values of the weights. Recall that beside classification accuracy, this is an additional objective performance measure used for the analysis of the quantized neural network.

**Figure 8:**
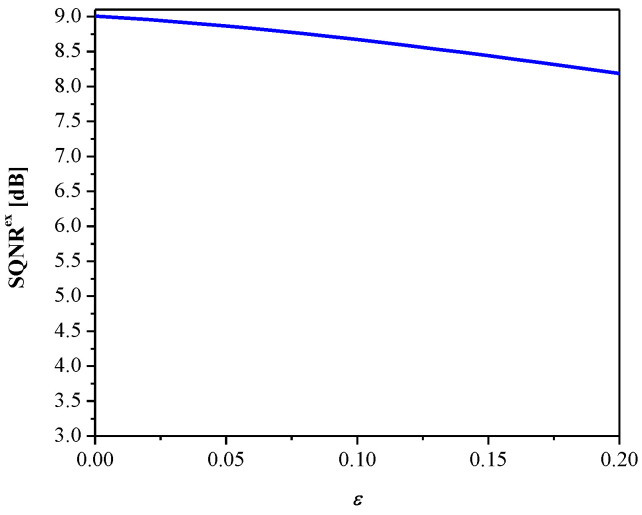
SQNR vs. *ε* achieved for weights quantization.

**Figure 9:**
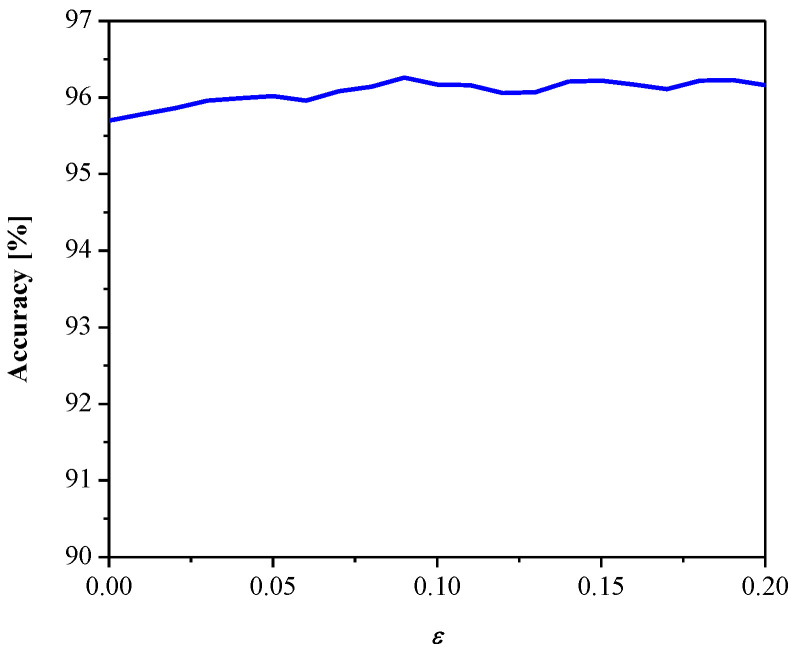
Performance of quantized MLP for different values of *ε*.

**Figure 10:**
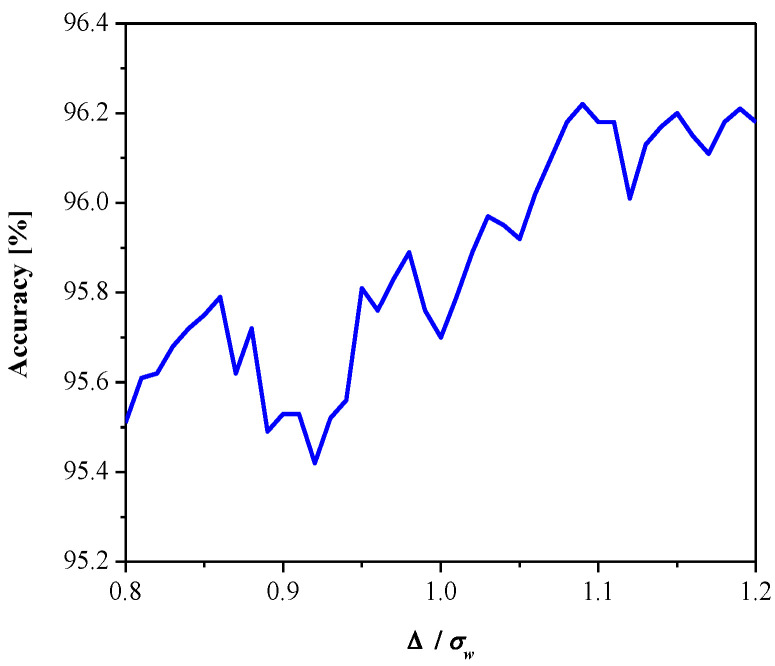
Classification accuracy of quantized MLP network as a function of quantization step size, *ε* = 0.09.

Figure 8 gives SQNR*^ex^* versus the parameter *ε*. It can be observed that SQNR decreases as *ε* increases, which is in accordance with the theoretical results presented in Figure 5 (observing one particular variance value). In addition, both the theoretical and experimental values of SQNR agree well (considering some specific value of *ε* for a given variance value). Moreover, we examined the influence of the parameter *ε* (observing the same range as in Figure 8) on the MLP performance obtained in the test data [41], as shown in Figure 9. Note that the increasing of *ε* slightly increases performance (classification accuracy), while the performance maximum is achieved for *ε* = 0.09. Thus, we can conclude that *ε* affects the introduced performance measures differently for the given network configuration and input data. Since classification accuracy is a relevant measure for neural networks, for the purpose of further analysis, we adopt corresponding values of classification accuracy and the SQNR achieved for *ε* = 0.09, which are listed in Table 1. In addition, we plot in Figure 10 the classification accuracy as the function of step size Δ/*σ_w_*, when *ε* = 0.09. It can be seen that the maximum score of classification accuracy is achieved for Δ = 1.09, which corresponds to the theoretically optimal value, confirming the applicability of the optimal quantizer.

Table 1 also summarizes the achieved performance (classification accuracy and SQNR) for adaptive 1-bit (binary) quantization of Laplacian source [26] and existing 2-bit solutions taken from [17,18,20,21], which serve as the baselines for comparison. The classification accuracy score of the non-quantized MLP network (full precision weights) is also included. Regarding the baseline 2-bit uniform quantizer [17], it is described by the following set (in a positive part) of representative levels {*y*_3_ = *w*_max_−Δ, *y*_4_ = *w*_max_} and by the set of decision thresholds {*x*_o_ = 0, *x*_1_ = Δ, *x*_2_ = 2Δ}, where $\Delta =2w_{\max}/2^{R}-1$ [17], *R* = 2, and *w*_max_ is the maximal value of the weights. For the 2-bit uniform quantizer defined in [18], it holds: {*y*_3_ = *w*_max_*^a^* − 3Δ/2, *y*_4_ = *w*_max_*^a^* − Δ/2} and {*x*_o_ = 0, *x*_1_ = Δ, *x*_2_ = 2Δ}, where $\Delta =2w_{\max}^{a}/2^{R}$ [18], *R* = 2, and *w*_max_*^a^* is the maximal absolute value of the weights. In the case of the 2-bit non-uniform quantizer described in [20], it holds: {*y*_3_ = Δ/2, *y*_4_ = 2Δ} and {*x*_o_ = 0, *x*_1_ = Δ, *x*_2_ = 3Δ *= x*_max_^opt^}, where $\Delta =2x_{\max}^{\mathrm{opt}}/3$ [20] and *x*_max_^opt^ denotes the value of the optimal support region threshold of the proposed 2-bit uniform quantizer. Finally, a 2-bit non-uniform quantizer [21] is defined as follows: {5/8 = *F*(*y*_3_), 7/8 = *F*(*y*_4_)} and {*x*_o_ = 0, 3/4 = *F*(*x*_1_)}, where $F\left(x\right)=1-\frac{1}{2}\exp\left(-\sqrt{2}x\right)$.

Observe in Table 1 that quantized MLP using the proposed adaptive 2-bit quantizer provides a classification accuracy score that is only 0.6% below the full precision case, while the network size is reduced by 16 times, which is significant. Note also that our proposal is able to outperform all introduced 2-bit baselines, as quantized MLP in that case attains higher classification accuracy scores at the same compression level, along with the significantly higher SQNR. This can be interpreted in a manner that the benefit is attained as the result of proper quantizer design, as the baseline quantizer approaches [17,18,20,21] can be considered as suboptimal for the given task. Thus, we report the following gains in SQNR (in dB) and classification accuracy (in %): 7.08 dB and 1.56% with respect to the baseline in [17], 7.52 dB and 1.77% with respect to the baseline in [18], 17.6 dB and 3.88% with respect to the baseline in [20], and 11.12 dB and 3.53% when compared to the baseline in [21]. Moreover, a gain in performance over the 1-bit solution from [26] is also notable (4.5 dB in SQNR and 5.1% for classification accuracy), which is achieved at the expense of a slightly lower compression level. 

**Figure 11:**
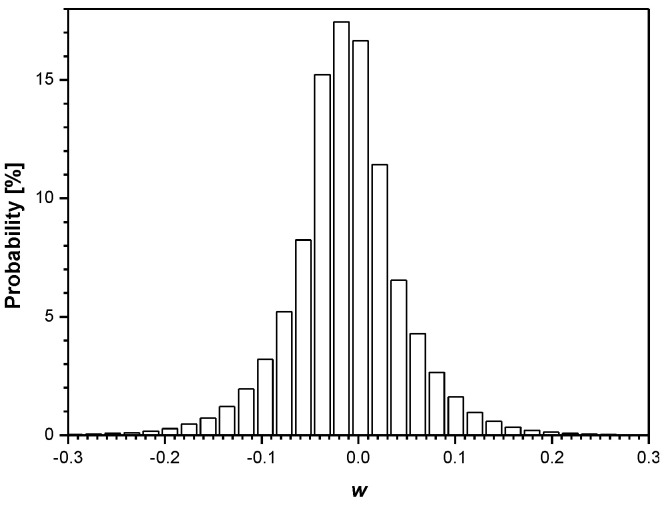
Distribution of fully connected layer weights of trained CNN network.

Additionally, we perform quantization of a simple CNN model [30] using the proposed 2-bit uniform quantizer. The model consists of one convolutional layer, one max-pooling layer, one fully connected layer, and the output layer. The number of output filters in the convolutional layer is set to 32, whereas it’s kernel size is 3 × 3. The size of the pooling window is set to 2 × 2. The fully connected layer with 100 units on top of it, which is activated by the ReLU activation function, is placed further, before the output layer. Dropout of 0.5 is performed on the fully connected layer. The network is trained for 10 epochs in batches of size 128 on the same MNIST dataset as the MLP model. The distribution of the weight coefficients in the fully connected layer after the training process is presented in Figure 11.

**Figure 12:**
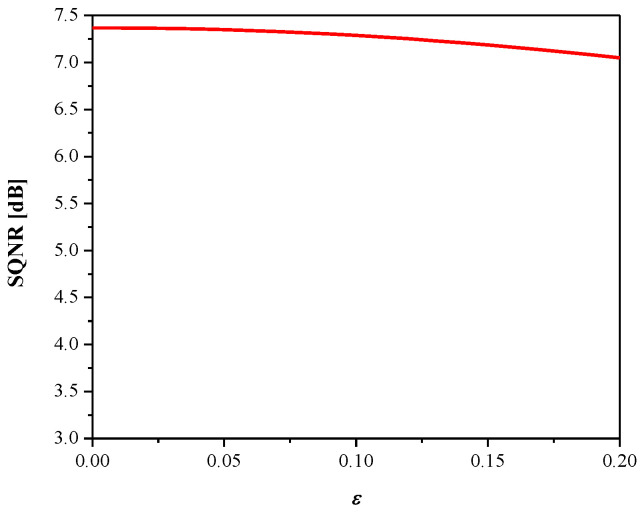
SQNR vs. *ε* achieved for weights quantization (CNN).

**Figure 13:**
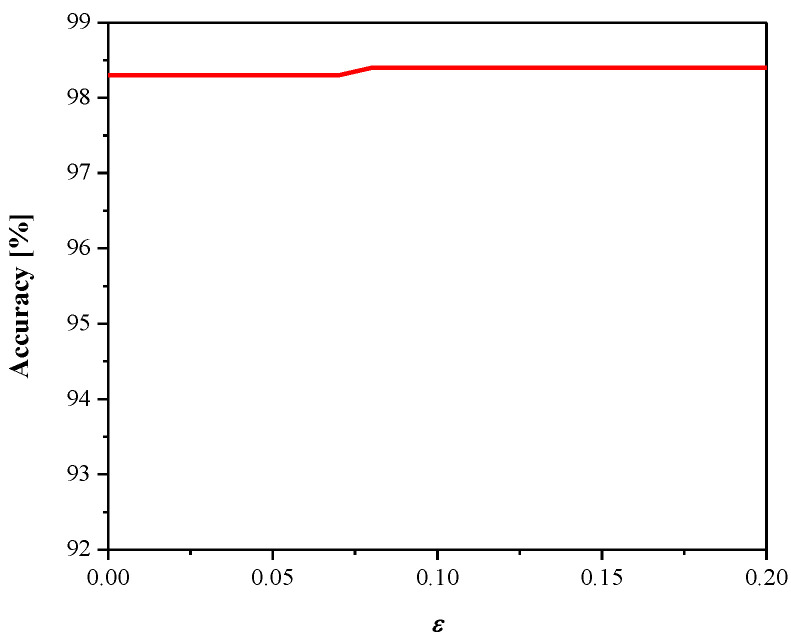
Performance of the quantized CNN model for various values of *ε*.

We obtained a classification accuracy of 98.7% in the test dataset, which is a higher accuracy compared to that of the MLP model and could be expected. The quantized CNN model is obtained by applying the proposed 2-bit quantizer (Δ = 1.09) for the task of fully connected layer weight quantization. Figure 12 and Figure 13 give the dependence of SQNR and classification accuracy on the parameter ε, respectively, where similar conclusions can be derived as in the previous case where MLP is considered (see Figure 8 and Figure 9). The achieved maximal classification accuracy of the quantized CNN model is 98.4%, achieved for ε = 0.08, which is only 0.3% less than the full-precision accuracy.

Finally, Table 2 compares the attained performance (classification accuracy and SQNR) of the quantized CNN in cases when the proposed (Δ = 1.09, ε = 0.08) and baseline (the same as in Table 1) quantization approaches are implemented. The superiority of the proposed quantizer is clearly visible from the given table, as significant improvements in SQNR and classification accuracy can be observed: 14.4 dB and 2.1% with respect to the baseline in [17], 11.33 dB and 1.5% with respect to the baseline in [18], 22.17 dB and 2.3% with respect to the baseline in [20], and 16.39 dB and 2.3% with respect to the baseline in [21]. Finally, better performance is also found in comparison to the 1-bit quantizer reported in [26].

Based on the overall analysis and results presented herein, we can point out that our proposal is very effective and is worth implementing for the post-training compression of neural networks. 

## 4. Conclusions

In this paper, a detailed analysis of 2-bit uniform quantization for processing the data described with the Laplacian PDF was conducted from both a theoretical and experimental point of view. During the theoretical design, using MSE distortion as a criterion, it was shown that distortion has a global minimum, specified by using the proposed iterative rule; thus, the optimal 2-bit uniform quantizer model was developed. In addition, the asymptotic value of the crucial designing parameter (step size) was provided, which is very close to the theoretically calculated optimal value. The analysis in a wide range of input data variances was also carried out, where a low robustness level and the need for adaptation (as an efficient method for performance improvement) were indicated. To obtain experimental results, the proposed adaptive model was employed in real-data processing using the parameters of a neural network (weights), where, as proof of concept, both MLP and CNN networks were used. It was demonstrated that the employed MLP and CNN in combination with the proposed approach (i.e., quantized neural networks) are able to achieve near-optimal performance, with significantly lower memory requirements when compared to MLP and CNN with full precision weights, which also lead to faster classification. Moreover, the advantage over different 2-bit quantizer solutions available in the literature, providing the same compression level, as well as the 1-bit quantizer solution, was demonstrated. Based on these promising results, one can expect implementation of the proposed quantizer in the compression of some modern networks, knowing that they are based on MLP, and also to IoT resource-constrained devices. Moreover, our future research will be directed toward the compression of some of the state-of-the-art networks, such as ResNet, AlexNet, or GoogleNet.

## Figures and Tables

**Table 1:** Performance (classification accuracy and SQNR) of quantized MLP for various applied quantization models.

				Quantizer			Full Precision
	1-Bit [26]	2-Bit Uniform [17]	2-Bit Uniform [18]	2-Bit Non-Uniform [20]	2-Bit Non-Uniform [21]	2-Bit Uniform Proposed	
Accuracy (%)	91.12	94.70	94.49	92.38	92.73	96.26	96.86
SQNR (dB)	4.25	1.63	1.19	−8.89	−2.41	8.71	-

**Table 2:** Performance (classification accuracy and SQNR) of quantized CNN for various applied quantization models.

				Quantizer			Full Precision
	1-Bit [26]	2-Bit Uniform [17]	2-Bit Uniform [18]	2-Bit Non-Uniform [20]	2-Bit Non-Uniform [21]	2-Bit Uniform Proposed	
Accuracy (%)	96.2	96.3	96.9	96.1	96.1	98.4	98.7
SQNR (dB)	3.21	−7.08	−4.01	−14.85	−9.07	7.32	-

## Data Availability

Data analyzed in this paper are available in a publicly accessible repository (MNIST dataset): http://yann.lecun.com/exdb/mnist/ (accessed on 15 May 2021).
